# Correspondence

**Published:** 1902-03

**Authors:** 


*Editor Journal-Record of Medicine :*

The *Cincinnati Lancet-Clinic* is right in censuring the pedantry of the old school societies to exclude from membership the "irregulars." It denotes ridiculous conceit in them to want to improve upon legislation. Since the privilege to represent a license has been taken from the sheepskins, the passing of the State examination should also qualify in other regards. Looking at this diploma-jealousy from a scientific standpoint and methodically, one cannot help, even, to discover considerable greenhorn-philosophy in it. Have the gentlemen who look down on an irregular as an inferior cast of practitioners, ever reflected upon the intrinsic virtue of a diploma? I doubt it. What is it? It is a certificate of the lowest degree in medicine, without which a student is not considered able to practice. Mind, *the lowest degree!* It must be presumed, therefore, that if, after years, a medical practitioner is so extremely proud of his diploma, he did not learn much since he was given it. He has nothing new to show up, and so he keeps on dancing under his mistletoe. The "doctor" is good enough, so far as that goes, as long as it is the only handle to the name. But if the world-wide renowned surgeon in Vienna used to display a shingle, "Billroth," and absolutely nothing else, it was justifiable pride. A letter with the name, Billroth, on the address, "in Europe," would have reached him, no matter where mailed in this country. Well, and is there anybody in the profession here, who will speak of "Dr. Sims?" Is it not an honoring of the man when the Dr. is dropped? Would the prefix not drag down the eminent master to the college-benches?

We may conclude, therefore, that as long as, in their scientific gatherings the medical practitioners are anxious to be ycleped "Doctors," and satisfied with it in their ambition, they must be considered bodies of very poor—students. E. T.

